# Royal jelly induces ROS-mediated apoptosis in acute lymphoblastic leukemia (ALL)-derived Nalm-6 cells: Shedding light on novel therapeutic approaches for ALL

**DOI:** 10.22038/IJBMS.2024.76261.16498

**Published:** 2024

**Authors:** Somayeh Yazdanparast, Davood Bashash, Amirsalar Nikkhah Bahrami, Mohammad Ghorbani, Mehrdad Izadirad, Mehdi Bakhtiyaridovvombaygi, Seyede Zahra Hasanpour, Ahmad Gharehbaghian

**Affiliations:** 1 Laboratory Hematology and Blood Bank Department, School of Allied Medical Sciences, Shahid Beheshti University of Medical Sciences, Tehran, Iran; 2 Laboratory Hematology and Blood Bank Department, School of Allied Medical Sciences, Mashhad University of Medical Sciences, Mashhad, Iran; 3 Laboratory Hematology and Transfusion Medicine, Department of Pathology, Faculty of Medicine, Gonabad University of Medical Sciences, Gonabad, Iran; 4 Laboratory Hematology and Transfusion Medicine, Department of Medical Laboratory Sciences, Faculty of Allied Medicine, Gonabad University of Medical Sciences, Gonabad, Iran; 5 Pediatric Congenital Hematologic Disorders Research Center, School of Medicine, Shahid Beheshti University of Medical Sciences, Tehran, Iran

**Keywords:** Acute lymphoblastic - leukemia (ALL), Apoptosis, Nalm-6, Reactive oxygen species - (ROS), Royal jelly (RJ)

## Abstract

**Objective(s):**

Until recently, a conventional chemotherapy regimen for Acute lymphoblastic leukemia (ALL) is considered an efficient therapeutic method in children. However, suboptimal long-term survival rates in adults, disease relapse, and drug-induced toxicities require novel therapeutic agents for ALL treatments. Today, natural products with pharmacological benefits play a significant role in treating different cancers. Among the most valued natural products, honey bees’ royal jelly (RJ) is one of the most appreciated which has revealed anti-tumor activity against different human cancers. This study aimed to evaluate anti-leukemic properties and the molecular mechanisms of RJ cytotoxicity on ALL-derived Nalm-6 cells.

**Materials and Methods:**

The metabolic activity was measured by MTT assay. Apoptosis, cell distribution in the cell cycle, and intracellular reactive oxygen species (ROS) level were investigated using flow cytometry analysis. Moreover, quantitative real-time PCR (qRT-PCR) was performed to scrutinize the expression of various regulatory genes.

**Results:**

RJ significantly decreased the viability of Nalm-6 cells but had no cytotoxic effect on normal cells. In addition, RJ induced ROS-mediated apoptosis by up-regulating pro-apoptotic genes while decreasing anti-apoptotic gene expression. The results outlined that ROS-dependent up-regulation of FOXO4 and Sirt1 inhibits the cells’ transition to the S phase of the cell cycle through p21 up-regulation. The qRT-PCR analysis of autophagy-related gene expression also demonstrated that RJ induced BECN1 mediated autophagy in Naml-6 cells.

**Conclusion:**

Taken together, this study showed that RJ can be utilized as a potent natural substance to induce ALL cells’ programmed cell death. However, further studies are required to examine this compound’s pharmaceutical application.

## Introduction

Acute lymphoblastic leukemia (ALL) is the clonal expansion of lymphoid hematopoietic progenitors in the bone marrow (BM), peripheral blood (PB), and extramedullary sites. Generally, 75–80% of ALL cases are B-ALLs, while the remaining cases are characterized as T-ALL (1). US incidence of ALL is estimated at 1.7 cases per 100,000, while worldwide incidence is estimated at 0.4 to 2 per 100,000 in 2020 (2, 3). Incidence of ALL decreases with age and follows a bimodal distribution, the first peak occurring in childhood and the second peak around 60 years of age (4). 

In the last 50 years, significant progress has been made toward treating ALL since it represents 25% of childhood cancers (5). Most conventional chemotherapy regimens for ALL patients include induction therapy, multi-agent consolidation, re-induction, and maintenance therapy. Moreover, these patients receive central nervous system (CNS) prophylaxis treatments and may undergo allogeneic hematopoietic stem cell transplantation (HSCT) (6). However, this intensive chemotherapy regimen, accompanied by long-term survival rates exceeding 90% in children, yielded unsatisfactory results in adults, along with only 35–45% long-term survival rates (7). The relapse rate for children and adults is 20% and 40–50%, respectively (3). Furthermore, these treatments have side effects such as cardiac, neurological, endocrine, social disorders, and secondary malignancies, which direct attention to designing more efficacious treatment approaches (8).

The natural compounds containing molecules with anti-tumor effects could present new treatment possibilities by targeting different cellular mechanisms (9). Royal jelly (RJ) as a natural honey bee product, is one of the milestones of this compound which has received increasing interest as an effective and safe treatment strategy in both traditional and modern medicine (10).

RJ is a milky and viscous substance secreted from worker honeybees’ hypopharyngeal and mandibular glands (*Apis mellifera L*), which is also well-known as the exclusive food for the queen bee and honeybee larvae development (11). RJ is a powerful supplement containing significant health-promoting and bioactive components such as proteins, sugars, lipids, vitamins, and amino acids. In addition, RJ has several physiological, biological, pharmacological, and organo-protective properties, including anti-oxidant, antimicrobial, wound healing, immunomodulatory, anti-aging, anti-hypercholesterolemia, and anti-hypertension (12). RJ has significant advantages in combating various malignancies such as breast, prostate, and ovarian cancers. Likewise, RJ offered to be a profitable nutrition for cancer therapy-induced toxicity reduction (13). The safety and efficacy of RJ have also been demonstrated in *in vivo* experiments (14–16). As a result of RJ and its main component’s unique potential for treating various kinds of cancer, it could be a combinatorial drug for improving human health.

Although many studies on human/animal models and various cancer cells have focused on the anti-cancer properties of RJ, there is very little information on the leukemic cells. Therefore, this study aimed, for the first time, to probe the cytotoxic effect and the underlying molecular mechanisms of RJ on ALL-derived Nalm-6 cells. Therefore, Nalm-6 cells were subjected to various concentrations of RJ. Then, metabolic activity, growth kinetics, and transcriptional alteration of reactive oxygen species (ROS)-, apoptosis-, autophagy- and cell cycle-related genes were investigated. Afterward, the RJ effect on L929 cells, peripheral mononuclear cells (PBMC), and red blood cells (RBCs) were examined to evaluate their safety. 

## Materials and Methods

***Chemicals and reagents***

Roswell Park Memorial Institute (RPMI) 1640, Dulbecco’s Modified Eagle Medium (DMEM), and fetal bovine serum (FBS) were provided by GIBCO (Gibco^TM^, USA). 3-(4,5-dimethylthiazol-2-yl)-2,5-diphenyl-2H-tetrazolium bromide (MTT) and complementary DNA (cDNA) synthesis kit were purchased from Thermo Fisher Scientific (USA). Dimethyl sulfoxide (DMSO) was bought from Sigma-Aldrich (Germany). L-Glutamine (Solution 100X), penicillin-streptomycin (Solution 100X) and trypsin Ethylenediaminetetraacetic acid (EDTA) were acquired from PAN Biotech (Germany). Annexin-V apoptosis detection kit was purchased from BioLegend (USA). DNA staining reagent kit and intracellular ROS detection kit were provided by Sigma-Aldrich (Germany). Trizol was procured from GeneAll RiboEx ^TM^ (Korea). Real Q Plus Master Mix Green was bought from Amplicon (Denmark). Ficoll was obtained from Lymphodex (Germany). Fresh RJ was supplied by local beekeepers in Khansar (33.2209° N, 50.3153° E), Iran, in the spring of 2022.

***Preparation of soluble RJ extract***

Fresh RJ was obtained from the bees mainly fed with Astragalus plants. RJ was collected from the bees’ larvae by the beekeeper and brought to our research laboratory in a dark glass container at -20 °C. First, the content of 10-hydroxy-2-decenoic acid (10-HDA), RJ major, and unique fatty acid, were evaluated by high-performance liquid chromatography (HPLC) to examine the RJ authenticity and quality (17). For this purpose, a sample of RJ was sent to the Hourtash laboratory (Njafabad, Isfahan). Based on the laboratory protocol mentioned in a previous study (18), “the RJ sample was defrosted at room temperature, then 1 ml of 0.03 mol/l hydrochloric acid, 2 ml water, and 30 ml HPLC grade ethanol were added to 0.5 gr of the sample and dissolved by ultra-sonication. After centrifugation at 3500 rpm for 10 min, the sample was filtered and analyzed. Five concentrations of 10-HDA, including 25, 50,100, 150, 200, and 250 mg/l, in HPLC grade ethanol were prepared to create the external standard curve. 10-HDA content of RJ was analyzed with a reversed-phase (RP) Agilent ZorbaxVR Eclipse C18 (2504.6 mm) column on an Agilent 1100 instrument coupled to a DAD detector (HP G1315A, Agilent Technologies, Palo Alto, CA, USA) at 210 nm, using a mobile phase composed of methanol, pure water, 0.03 mol/l hydrochloric acid (55: 45: 5 v/v/v) in the isocratic mode. The injection volume and flow rate were set at 5 μl and 1 ml/min. Separation was performed at a column temperature of 25 °C” (18). Then, the RJ stock solution was prepared as previously described (19). Briefly, 10 g of native RJ was dissolved in 20 ml of PBS, and the initial stock was prepared at a concentration of 500 mg/ml, centrifuged at 12000 rpm for 30 min at 4 °C (Hettich, Germany), and separated into three layers. The top clear layer was pooled and sterilized using 0.22 μm Syringe filters (JetBiofill, Spanish) and stored at a -80 °C freezer until use. Finally, the supernatant was diluted with the culture medium to various working concentrations.

***PBMC isolation***

Human PBMCs were isolated using Ficoll-Paque density gradient centrifugation. Initially, PBMC was collected from a healthy donor using EDTA tubes and diluted with an equal volume of serum-free medium. Then, Ficoll-Paque was added at the bottom of a new tube without touching the tube side, and diluted cell suspension was pipetted on top of Ficoll-Paque without intermixing (10 ml of Ficoll-Paque for 20 ml of diluted blood) and centrifuged at 2000 rpm for 30 min. Next, the resulting layer of PBMC (the barrier between Ficoll and plasma) was carefully transferred to a new tube to wash twice with PBS at 1500 rpm for 10 min to remove platelets. The obtained PBMC plate was finally suspended in 1 ml complete media (as defined in the next section) and used for desired experiments.

***Cell culture and treatment***

Acute Lymphoblastic Leukemia cell line (Nalm-6) and normal mouse fibroblast cell line (L929) were purchased from the Pasteur Institute collection (Tehran, Iran). Human PBMCs were isolated by the Ficoll-Paque method. The Nalm-6 and PBMC routinely were cultured in RPMI-1640 medium supplemented with 2 mM L-glutamine, 10% heat-inactivated FBS, 100 IU/ml penicillin, and 100l g/L (PBMC were maintained in 20% FBS). L929 fibroblasts were propagated in DMEM medium enriched with 2mM L-glutamine, 10% FBS, 100IU/ml penicillin, and 100lg/L streptomycin. The cell cultures were incubated in a standard cell culture environment and a humidified atmosphere containing a 5% CO_2_ incubator at 37 °C (Memmert, Germany) and kept in the logarithmic phase. Then, all cells were treated with designed concentrations of the RJ stock solution (1, 1.5, 2, 2.5, 3, and 3.5 mg/ml).

***Cell cytotoxicity assessments***

*In vitro* assessment of the cytotoxicity effect of RJ towards cancer and normal cells was investigated by conventional colorimetric MTT assay to assess the reduction of yellow MTT solution into dark purple formazan crystal. We first tested a wide range of RJ concentrations from 1 μg/ml to 100 mg/ml on Nalm-6 cells based on other studies (19–21), and repeating this evaluation showed that the effect of this substance at a dose of 1 mg/ml starts 24 hr after exposure to Nalm-6 cells, which significantly increases comparable to the control after 48 and 72 hr. Then cells were seeded in 96-well plates (JetBiofill, Spanish) at the density of 12×10^3^ (for Nalm-6), 4×10^3^ (for L929), and 250×10^3^ (for PBMC) per well and treated with 1, 1.5, 2, 2.5, 3, and 3.5 mg/ml of RJ for 24 and 48 hr (Nalm-6 for 24, 48, and 72 hr). Then, 10 μl of MTT reagent (5 mg.ml^-1^ in PBS) was added to each well, and the plates were incubated at 37 °C for 4 hr. In the next step, the plate was centrifuged at 4000 rpm for 15 min to remove the supernatant (L929 fibroblasts do not require centrifugation). Then, 100 μl DMSO was added to dissolve e formazan crystals. An enzyme-linked immunosorbent assay (ELISA) reader (BioTek ELx800, USA) was used to measure optical densitometry (OD) of the resulting formazan purple crystals solubilized in DMSO after 15 min at 37 °C at a wavelength of 570 nm. Finally, the percentage of cell viability was calculated by the following formula (Metabolic activity (%) = [OD _Treated cells_/ OD _Control cells_] × 100). The assays were performed in triplicates and at least three times.

***Calculation of IC***_50_ ***values***

Each agent’s half maximal inhibitory concentration (IC_50_) value is also called its most potent inhibitory concentration. The data from cell viability assessments are analyzed by relevant software to calculate the IC_50_ value, and then the RJ concentrations are plotted against cell viability. Accordingly, 48 hr MTT results were processed using GraphPad Prism software version 9 to determine the IC_50_ value of RJ against Nalm-6 cell lines (GraphPad Software, San Diego, CA, USA), and the IC_50_ value is determined by non-linear regression.

***Flow cytometric assessments of apoptosis***

The flow cytometry technique was carried out to evaluate the RJ implication of inducing apoptosis using annexin V-propidium iodide (PI) co-staining. Nalm-6 cells were seeded at a concentration of 140×10^3^ cells per well on a 12-well cell culture plate (JetBiofill, Spanish) and subjected to various concentrations of RJ (1.5, 2.5, and 3.5 mg/ml). Then, the cells were harvested after 48 hr treatment, rinsed with PBS, and re-suspended in 100 μl of the incubation binding buffer. Next, the cells were stained using an Annexin V-FITC/PI apoptosis detection kit following the manufacturer’s instructions, and the results were calculated using the FlowJo^TM^ software version 7 (FlowJo™ Software, BD Life Sciences, USA). Annexin V-positive and PI-negative cells were considered in the early apoptotic phase, and cells with positive staining both for Annexin-V and PI were consumed to undergo late apoptosis. Annexin-V negative and PI-positive cells were quantified as necrotic cells. Flow cytometry was also performed on PBMC and L929 cells to investigate RJ’s apoptotic effect on normal cells (at least 10000 cells were required).

***Flow cytometric assessments of cell cycle***

PI staining was considered to investigate RJ’s impact on cellular DNA content and cell distribution analysis. Nalm-6 cells were seeded into a 12-well cell culture plate at 140×10^3^ cells per well and incubated with several concentrations of RJ (1.5, 2.5, and 3.5 mg/ml) for 48 hr. Following cell collection, cells were washed twice with cold PBS and fixed in 70% ethanol in the dark on ice. The next day, the cells were exposed to RNAse for 30 min at 37 °C after being washed with ice-cold PBS. Later, fluorochrome-conjugated PI was used to stain the DNA of these cells for 30 min at 4 °C in the dark. Finally, cellular DNA content was quantified using a flow cytometer (at least 10,000 cells were required). FlowJo^TM^ software version 7 was used for interpreting the obtained data.

***Detection of intracellular ROS***

The potential of RJ in producing ROS inside the cells can be measured by 2′,7′-dichlorofluorescein diacetate (DCFAD) staining to analyze ROS activity within the cell. DCFAD is oxidized to a fluorescence dye 2′,7′ - dichlorofluorescein (DCF) in live cells. Nalm-6 cells were pretreated with several doses of RJ (1.5, 2.5, and 3.5 mg/ml) for 48 hr in a 12-well cell culture plate with a cell density of 140×10^3^ cells per well. The cells were washed with PBS and re-suspended in PBS containing DCFAD for 30 min at 37 °C in the incubator. Finally, DCF fluorescence intensities of the samples were detected using a flow cytometer (at least 10000 cells were required). The data analysis was performed by FlowJo^TM^ software version 7.

***RNA extraction and cDNA synthesis***

RNA extraction and cDNA synthesis were the first steps to assess the alternation in mRNA expression of selected genes in the Nalm-6 cell line. Hence, control cells and cells treated for 48 hr with 1.5 and 2.5 mg/ml of RJ concentration on a 6-well cell culture plate (JetBiofill, Spanish) were collected, and their total RNA was extracted using the Trizol method. A Nanodrop TM 2000 Spectrophotometer (Thermo Fisher Scientific, USA) and 1% agarose gel electrophoresis were used to analyze RNA quantity and integrity, respectively. Then, 1 µg total RNA was reveres-transcribed with a cDNA synthesis kit following incubation for 5 min at 25 °C, followed by 60 min at 42 °C. The reaction was terminated by heating for 5 min at 70 °C. Finally, the synthesized cDNA was stored at -20 °C until being used.

***Quantitative real-time PCR (qRT-PCR)***

The mRNA expression of ROS-, apoptosis-, autophagy- and cell cycle-related genes were determined by qRT-PCR. The qRT-PCR reaction was performed using Rotor-Gene Q (Qiagen, Germany) in a total volume of 15 μl including 4.5 μl Real Q Plus Master Mix Green, 2 μl of template cDNA, 1 μl of forward and reverse primers, and 7.5 μl of nuclease-free water. Table 1 presents primer sequences for desired genes. Adapted thermal cycling conditions included an initial activation step at 95 °C for 15 min, followed by a total of 40 cycles (denaturation step at 95 °C for 10 sec combined with an annealing step at 58-63 °C for 15 sec and an elongation step at 72 °C for 15 sec). A melting curve analysis was carried out to verify the products’ specificity. The mean C_t_ of the target genes was analyzed by repeating these actions three times. As a final step, the fold change values were determined relative to the control gene using the Livak formula (2^-^^ΔΔCT^), adjusting the housekeeping gene. ABL1 was used as an endogenous reference gene to normalize all qRT-PCR data.

***RBC hemolysis assay***

A hemolysis assay is essential to examine the toxicity of RJ in contact with human blood. The hemolytic activity of RJ was investigated according to Mohammadlou *et al.* (27). Five milliliter of O^+^ of fresh blood, taken from a healthy donor, was collected in a heparinized tube and centrifuged at 1500 rpm for 10 min. After removal of the plasma, the RBC pellet was rinsed with cold PBS (pH: 7.4) at 1500 rpm for 5 min and mixed by inversion (centrifuging and washing steps were repeated two times). After the last washing step, RBC was diluted in PBS, and 0.5% suspension was provided. The prepared suspension was treated with 1.5, 2.5, and 3.5 mg/ml of RJ. The normal saline and deionized water were consumed by negative (0% hemolysis) and positive control (100% hemolysis), respectively. After mixing gently, each tube was incubated at 37 °C for 30 min and centrifuged at 1500 rpm for 10 min. Each sample’s supernatant was transferred into a cuvette, and spectrophotometers (Halo VIS-20, UK) were used to determine hemoglobin absorbance at 540 nm. Finally, the percentage of hemolysis is obtained by the following Equation.

Hemolysis (%) = [(OD _(Test)_ – OD _(Negative control)_) / (OD _(Positive control)_ – OD _(Negative control)_)] × 100 

***Statistical analysis***

Experimental data are expressed by the mean standard deviation (SD) to compare the mean values of three independent experiments performed in triplicate. One-way ANOVA (Turkey’s honestly significant difference criteria) was considered for statistical analysis. The descriptive statistics of the results were calculated in GraphPad Prism software version 9 (GraphPad Software, San Diego, CA, USA) and Microsoft Office Excel 2019 (Microsoft, Redmond, WA, USA). The statistical significance for the difference of means was assigned into one of two categories: *P*>0.05 (not significant) and *P*<0.05 (*significant). 

## Results

***Characterization of RJ authenticity and quality***

Authentication and quality of RJ were critical after preparation. In this vein, 10-HDA was examined as the significant marker of authenticity and quality of RJ by HPLC. Based on laboratory reports, RJ contains 3.0 % of 10-HAD (RJ grade based on 10-HAD content: A: >3.1, B: 2.1-3.0, C: 1.7-2.0, D: 1.4-1.6). Therefore, RJ used in this study has high quality and complies with the standards. 

***Time- and concentration-dependent inhibitory effects of RJ on NALM-6 cell survival***

**Figure 1:**
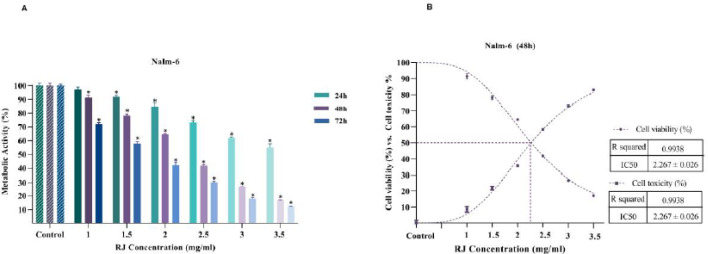
The effect of royal jelly (RJ) on the metabolic activity of Nalm-6 cells

The metabolic activity of treated cells with a designed concentration of RJ was examined with the MTT colorimetric assay. The results showed that RJ significantly hampers the metabolic activity of Nalm-6 cells in a time and concentration-dependent manner. As shown in Figure 1A, the survival rates of Nalm-6 cells after 24 hr incubation with RJ are 97, 91, 85, 73, 61, and 55% at doses of 1, 1.5, 2, 2.5, 3, and 3.5 mg/ml, respectively, while this percentage is respectively 91, 78, 64, 41, 26, and 16% at 48 hr and 71, 57, 42, 30, 17, and 12% at 72 hr. The IC_50_ valve of RJ on Nalm-6 cells was 2.2670.026 mg/ml after 48 hr treatments (Figure 1B). 

***RJ induced apoptosis and increased cell population in the Sub-G1 phase***

**Figure 2:**
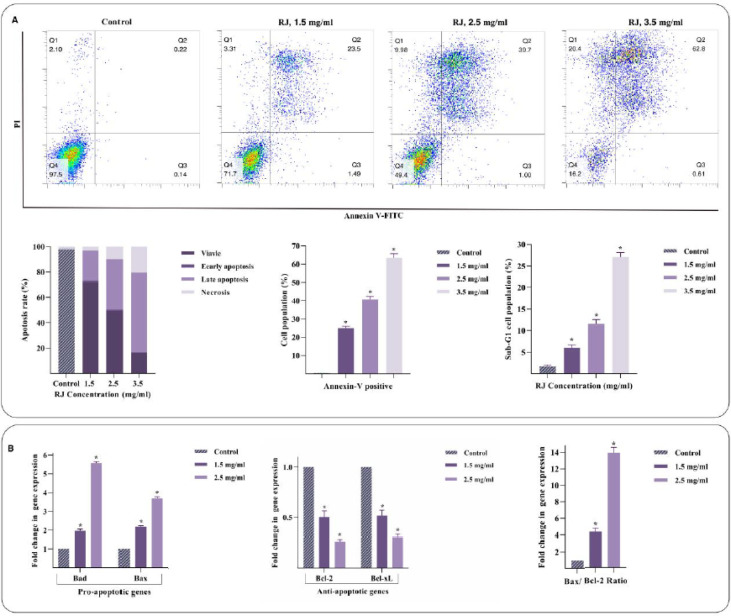
Effects of royal jelly (RJ) on apoptosis induction in Nalm-6 cells

Annexin-PI staining was performed to assess the pro-apoptotic activity of RJ in Nalm-6 cell lines. As shown in Figure 2A, the apoptotic rates (early and late) were respectively 24.99, 40.7, and 63.41 %, 48 hr after treatment with 1.5, 2.5, and 3.5 mg/ml of RJ. Consequently, RJ dose-dependently augmented the percentage of apoptotic Nalm-6 cells by comparing untreated cells, which agreed with the enhanced hypodiploid cell population in the Sub-G1 phase. In follow-up experiments, the mRNA expression level of a panel of apoptotic-related genes was evaluated using qRT-PCR to ascertain the molecular pathways through which RJ induces its apoptotic effect. According to the results shown in Figure 2B, RJ induces mitochondria-mediated apoptosis through increased levels of Bax and Bad, but not Bcl-2 and Bcl xL, and up-regulates the Bax/Bcl-2 ratio. 

***RJ inhibited Nalm-6 proliferation***

**Figure 3:**
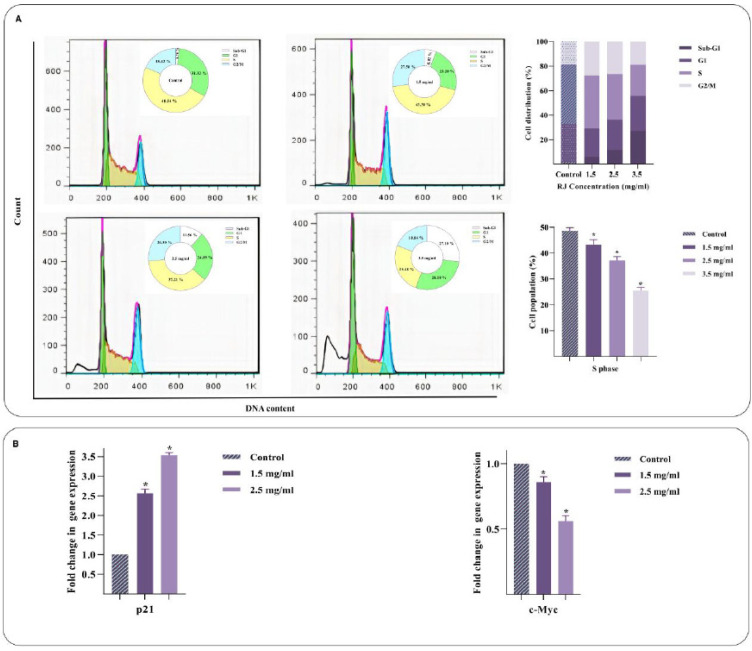
Effect of royal jelly (RJ) on cell cycle progression of Nalm-6 cells

PI staining was implemented to examine the effect of RJ on the cell cycle progression of Nalm-6 cells after 48 hr 

exposure to different doses of this component. As shown in Figure 3A, RJ resulted in a significant reduction of cell population in the S phase and halted the ability of ALL cells to replicate in a concentration-dependent manner. The expression of p21 and c-Myc, crucial gene regulators of the transition from G1 to the S phase, was investigated to study the plausible mechanisms by which RJ provokes a growth-suppressive effect. p21, as a cyclin-dependent kinase inhibitor, impeded cell cycle progression, while c-Myc, known as a p21 inhibitor, fostered cell cycle progression (28, 29). Molecular analysis data revealed an apparent reduction in c-Myc expression coupled with up-regulation of p21 in response to RJ (Figure 3B). 

***RJ increased intracellular ROS generation***

**Figure 4:**
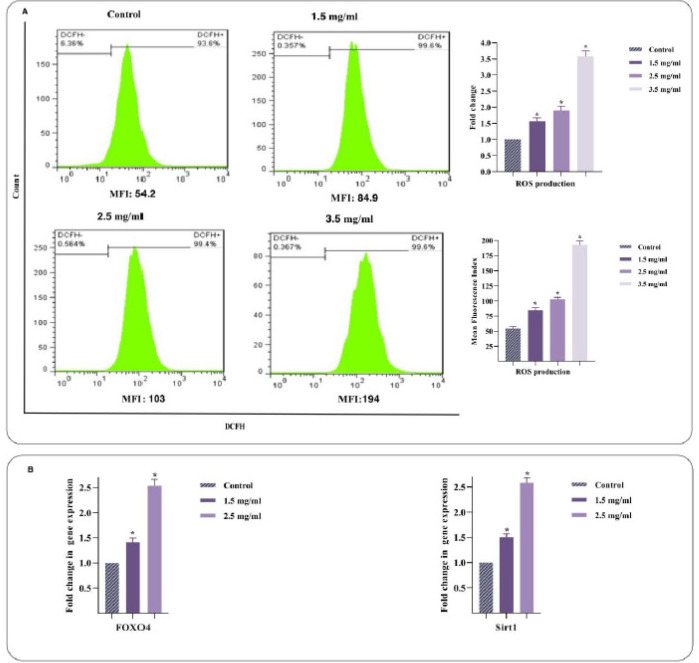
Effect of royal jelly (RJ) on ROS production in Nalm-6 cells

DCFDA staining was applied in treated cells for 48 hr to discover the potential of RJ in ROS accumulation inside the cells. As shown in Figure 4A, the ROS level in Nalm-6 cells was elevated with increasing RJ concentration. Thus, the levels of ROS were 1.5, 1.9, and 3.5 times more than control in 1.5, 2.5, and 3.5 mg/ml of RJ, respectively. The expression of FOXO4 and Sirt1 genes involved in ROS production was raised in RJ-treated cells compared to the control counterpart following the up-regulated ROS (4B). The data postulated that RJ mediated apoptotic cell death by augmentation of intracellular ROS levels in ALL-derived Nalm-6 cells.

***RJ-induced apoptosis was coupled with increased autophagy-related gene (ATG) expression***

**Figure 5:**
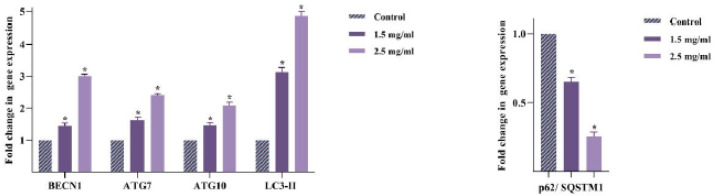
Effect of royal jelly (RJ) on the autophagy-related gene expression in Nalm-6 cells

This study aimed to assess the autophagy system’s effect on the anti-leukemic effect of RJ in light of the apoptotic property of RJ. The qRT-PCR was used to assess the expression of BECN1, ATG7, ATG10, protein one light chain 3-II (LC3-II), and p62/SQSTM1 as genes involved in autophagy machinery. Analyzing the gene expression results in treated Nalm-6 cells after 48 hr revealed that the expression of all these genes except p62/SQSTM1 was increased in Nalm-6 cells compared to the untreated group (Figure 5). The p62/SQSTM1 mRNA expression decreased, consistent with the autophagy activation. Therefore, autophagy activation could probably affect the cytotoxicity induced by RJ. Nevertheless, investigation on Nalm-6 cell lines was continued by treating cells with chloroquine (CQ) as an autophagy inhibitor concerning autophagy’s double-edged sword role. 

***RJ had no significant effect on normal cells***

**Figure 6:**
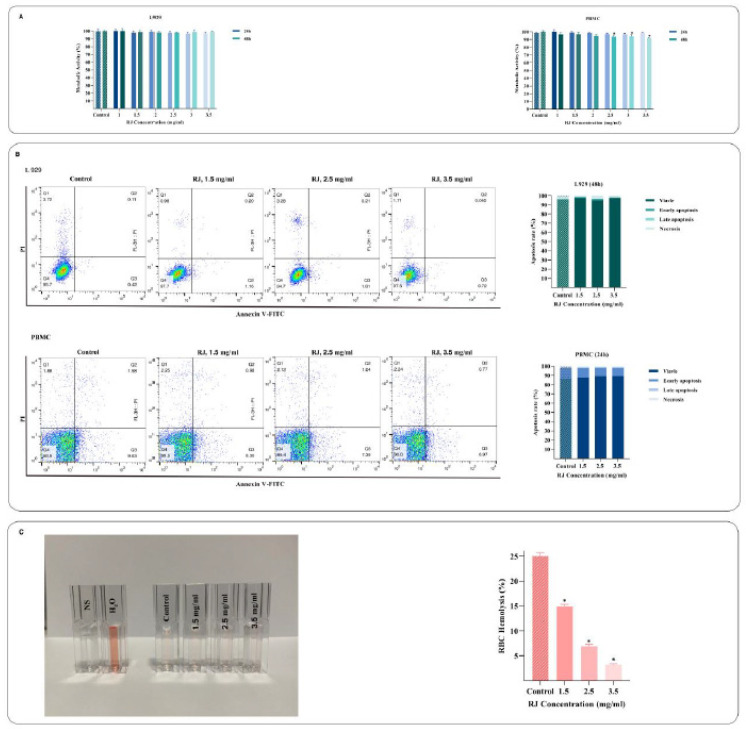
Effect of royal jelly (RJ) on normal cells. L929 and PBMC were used as models of normal cells. The MTT assay indicated that RJ has no cytotoxic effect on L929 and PBMC cells (A). Analysis of apoptosis using Annexin-V/PI staining and flow cytometry revealed that RJ has no significant apoptotic effect on normal cells (B). Hemolytic activity evolution suggested that the hemolysis rate decreases when RJ concentration increases (C). The values are given as the mean SD of three independent experiments. *P≤0.05 represents significant statistical changes from the control sample

L929 and PBMC cells were chosen as non-tumorous cells to determine whether RJ could inhibit the proliferative capacity of normal cells. The MTT assay revealed that RJ has a minimal cytotoxic effect on L929 and PBMC cells (Figure 6A). Furthermore, the apoptosis effect of RJ on L929 and PBMC cells was examined using flow cytometry analysis. As shown in Figure 6B, RJ has no apoptotic effect on normal cells. Moreover, the evaluation hemolytic activity of RJ indicated that the hemolysis rate decreases in RBCs incubated with increasing concentrations of RJ (Figure 6C). 

**Table 1:** Primer sequences used for qRT-PCR

Ref.	**Reverse primer (5'->3')**	**Forward primer (5'->3')**	**Accession number**	**Gene name**
(22)	CACTCAGACCCTGAGGCTCAAAG	CTGAAGCTGGTGGGCTGCAAATC	NM_005157.6	ABL
(22)	CCCATCCCTTCGTCGTCCT	CCCAGAGTTTGAGCCGAGTG	NM_004322.3	Bad
(22)	GTGGGCGTCCCAAAGTAGG	CGAGAGGTCTTTTTCCGAGTG	NM_001291428.2	Bax
(22)	ACAAATGCATAAGGCAACGATCC	GTGAAGCAGAAGTCTGGGAATCG	NM_000633.3	Bcl-2
(23)	CCTGAATGACCACCTAGAGCCTT	TGCATTGTTCCCATAGAGTTCCA	NM_001012270.2	Bcl-xL
(22)	GCGTTTGGAGTGGTAGAAATCT	CCTGTCACTGTCTTGTACCCT	NM_000389.5	p21
(22)	GCAGGATAGTCCTTCCGAGTG	CCACAGCAAACCTCCTCACAG	NM_001354870.1	c-Myc
(24)	CCCCTCCGTGTGTACCTTTTC	CACGTATGGATCCGGGGAAT	NM_001170931.2	FOXO4
(25)	ACAGCTTCACAGTCAACTTTGT	TAGCCTTGTCAGATAAGGAAGGA	NM_001142498.2	Sirt1
(22)	GCTCCTCTCCTGAGTTAGCCT	TGCAGGTGAGCTTCGTGTG	NM_001313998.2	BECN1
(22)	AGGCTCAGCCATGATGTGAT	CCCAGCAGGAACATCCAATA	NM_001131028.2	ATG10
(22)	GATGGAGAGCTCCTCAGCA	ATTGCTGCATCAAGAAACCC	NM_001136031.3	ATG7
(26)	TGAGATTGGTGTGGAGACGCT	ACGATACAAGGGTGAGAAGCAGC	NM_022818.5	LC3-II
*	GGGAGAGGGACTCAATCAGC	ACAGATGCCAGAATCCGAAGG	NM_001142298.2	p62/SQSTM1

Nalm-6 was treated with several concentrations of RJ for 24, 48, and 72 hr and MTT assays evaluated metabolic activity. As shown, RJ significantly decreased the viability of Nalm-6 cells in a concentration- and time-dependent manner (A). In addition, RJ caused a 50% reduction in Nalm-6 cell proliferation (IC_50_) in 2.267±0.026 mg/ml concentration (B). The values are given as the mean SD of three independent experiments.*P≤0.05 represents significant statistical changes from the control sample

Nalm-6 cells were pretreated with the desired concentration of RJ for 48 hr, and then apoptosis was determined by Annexin-V and PI staining. RJ vigorously increased the percentage of apoptotic cells in a dose-dependent way. Likewise, RJ augmented the cell population in the sub-G1 phase (A). Gene expression analysis revealed that RJ induced apoptosis by up-regulating pro-apoptotic genes while decreasing anti-apoptotic gene expression (B). The values are given as the mean SD of three independent experiments. *P≤0.05 represents significant statistical changes from the control sample.

Nalm-6 cells were pretreated with the desired concentration of RJ for 48 hr. The flow cytometry analysis using PI staining revealed that RJ diminished the population of cells in the S phase (A). The molecular analysis determined that the expression of p21 as the most critical S phase regulatory gene was increased, while oncogenic c-Myc expression decreased (B). The values are given as the mean SD of three independent experiments. *P≤0.05 represents significant statistical changes from the control sample

Nalm-6 cells were pretreated with various concentrations of RJ, and the ROS level was measured by DCFDA staining after 48 hr. RJ significantly increased ROS production in Nalm-6 cells in a concentration-dependent manner (A). The gene expression analysis showed the expression of FOXO4 and Sirt1 genes raised after treatment (B). The values are given as the mean SD of three independent experiments. *P≤0.05 represents significant statistical changes from the control sample.

ROS: reactive oxygen species

Nalm-6 cells were pretreated with several concentrations of RJ for 48 hr. Then, qRT-PCR was applied to analyze the selected gene expression. RJ led to increased expression of BECN1, ATG7, ATG10, and LC3-II genes, and RJ down-regulated p62/SQSTM1 expression. The values are given as the mean SD of three independent experiments. *P≤0.05 represents significant statistical changes from the control sample.

**Figure 7:**
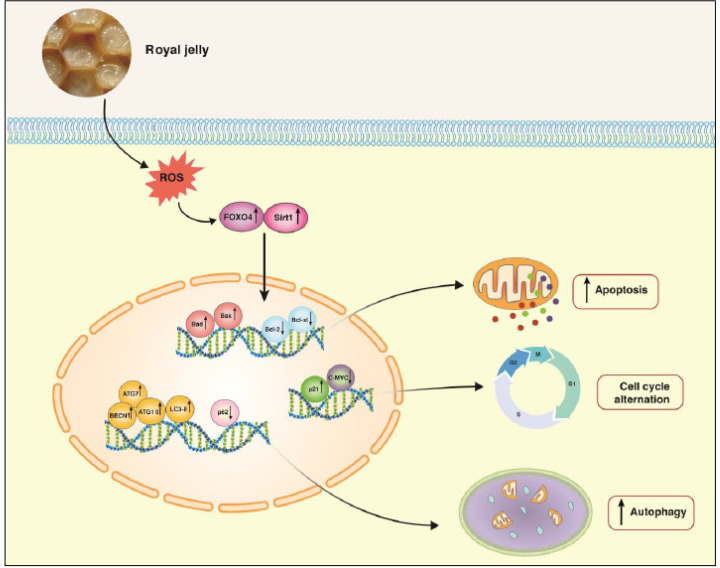
Schematic representation proposed for the plausible mechanisms of action of royal jelly (RJ) in Nalm-6 cells

Treatment of ALL-derived cells with RJ leads to ROS-mediated apoptosis, cell cycle alternation, and autophagy activation.

## Discussion

ALL is the most common subtype of leukemia in pediatricsand is characterized by abnormal proliferation of immature lymphocytes in the BM (30). Although significant improvements in childhood ALL treatment have been achieved, the unsatisfactory outcomes in older patients, disease relapse, and toxicities related to conventional treatments in all patients require new therapeutic opportunities for ALL (3, 7, 8). Natural products are critical in discovering anti-cancer drugs such as vincristine, irinotecan, paclitaxel, and etoposide. There has been an increase in interest in natural product-based treatments for ALL recently (31, 32). The Honey bee products such as RJ are among the most interesting in this field. Accumulating *in vivo *and* in vitro* studies have highlighted RJ’s anti-cancer properties (13). This study aimed to unravel the anti-leukemic effect of RJ. Although this study is investigating the anti-cancer activity of RJ against the Nalm-6 human ALL cell line for the first time, previous studies have declared that RJ exerts potent anti-cancer activities against various cancer types, including bladder, breast, colon, lung, and prostate cancer (19, 21, 33–35). 

Our data indicated that RJ significantly diminished the metabolic activity of the Nalm-6 cell line in a concentration and time-dependent way. Other studies have revealed the cytotoxicity of RJ against 5637 bladder cancer cells and PC3 prostate cancer cells (19, 21). The cell selective effect of RJ treatment can be noticed since RJ exhibited no cytotoxic effect on human PBMC and normal murine fibroblast cell lines. Accordingly, previous studies confirmed that RJ not only does not harm PBMC but also enhances their proliferation and strengthens the immune system (36). Another study by Vanesa *et al.* exhibited that bee product mixtures had a slightly cytotoxic effect on normal cells (37). Moreover, the hemolytic activity of RJ was measured to confirm the safety of this compound. RJ decreases the hemolysis rate, and the lowest amount was observed in doses higher than IC_50_ compared to the control. A study indicated that six-month ingestion of RJ in humans positively affects erythropoiesis and improves RBC production (38). Therefore, RJ is safe for normal cells and has no detrimental effect as an anti-leukemic agent. 

Preceding studies have established that the cytotoxicity of RJ can be attributed to free radicals and ROS generation (34, 35). ROS are oxidant molecules with a significant role in numerous biological functions such as cell cycle, proliferation, apoptosis, and signal transduction (39). According to Kamiya *et al.*, the prooxidant activity of RJ may be due to its fatty acid fraction, such as 10-HDA. In this study, 4-Hydroperoxy-2-decenoic acid ethyl ester (HPO-DAEE), a 10-HDA derivative, produced ROS in A549 cells (40). In this study, we demonstrated that Nalm-6 pretreatment with RJ resulted in a rise in ROS level in flow cytometric analysis. Therefore, the cytotoxic properties of RJ can be related to its prooxidant activity. In contrast, another study demonstrated that RJ induces anti-oxidant activity toward human lymphocytes, as well as enhancing cell proliferation and lifespan and lowering apoptosis (41). Hence, the hypothesis is that RJ enhances immune system function while combatting leukemic cells through modulating ROS generation.

Apoptosis is a form of programmed cell death through three signaling pathways, including mitochondrial, death receptor, and endoplasmic reticulum pathways (42). ROS promotes the outer mitochondrial membrane permeabilization and subsequent release of pro-apoptotic factors such as cytochrome c into the cytosol. Thus, ROS accumulation can initiate the mitochondrial pathway (43). Furthermore, ROS-mediated apoptosis may be related to its ability to activate the Forkhead box O (FOXO) proteins family. The FOXO family is a group of tumor suppressor transcription factors that regulate a wide range of biological activities inside the cell, including oxidative stress, cell cycle progression, and apoptosis in cancer cells (44). Several indications of apoptosis were analyzed in this research to understand the mechanisms involved in RJ-induced Nalm-6 cell apoptosis.

First, the flow cytometric examination demonstrated that RJ probably increases the apoptosis of Nalm-6 cells. It is worth mentioning that evaluation of the apoptotic effect of RJ in normal cells showed that this substance has no significant impact on normal cells, unlike cancer cells. The apoptosis process is highly regulated by a complex network of anti- and pro-apoptotic proteins called the BCL-2 protein family (42). Therefore, BCL-2 protein family expression was examined to ascertain the apoptosis induction. Based on reports, the lipid fraction of RJ can trigger overexpression of Bax, Cyt-c, p53, and caspase-3 while down-regulating Bcl-2 expression in colon cancer cells (34). Consistent with this finding, research found that 10-HDA can lead to elevations in Bax expression in lung cancer-derived A549 cells (35). Likewise, the present study showed that the expression levels of Bad and Bax (pro-apoptotic genes) were augmented, whereas the expression of Bcl-2 and Bcl-xl (anti-apoptotic proteins) declined. Furthermore, RJ distributed the balance between Bax/Bcl2, which favors increased apoptosis. 

Interestingly, apoptosis-related gene changes were accompanied by increased expression in oxidative stress-regulating genes, including Sitr1 and FOXO4. Sirt1 (a NAD-dependent protein deacetylase) is a member of the Sirtuin family, which regulates the expression of a wide range of genes. FOXO family genes such as FOXO4 are among the substrates of this protein, and Sirt1 increases the expression of this gene after binding and deacetylating (45). FOXO4 triggers apoptosis by increasing the expression of Bim, Cytochrome c, Caspase3, and Bax and down-expression of Bcl-2 (46). Thus, ROS production could up-regulate Sirt1 and FOXO4, initiating mitochondrial apoptosis via alteration in apoptotic-regulating genes. In particular, N-acetylcysteine (NAC), a potent anti-oxidant, prevented cell death triggered by RJ. Therefore, ROS generation is involved in the apoptosis process (40). 

Additionally, the increasing FOXO4 and Sirt1 could interact with p21 and p27 cyclin-dependent kinase inhibitors, leading to cell cycle alternation (44, 47). Thus, the results illustrated that up-regulation of Sirt1 and FOXO4 hinders the transition of cells from G1 to S phase of the cell by up-regulation of p21, which was confirmed by PI staining. In contrast, Nalm-6 cell pretreatment with RJ led to c-Myc gene down-expression. The c-Myc is an oncogenic protein that inhibits p21 and p27 gene expression, resulting in cell cycle progression (29). Therefore, c-Myc down-regulation fosters RJ antiproliferative capacity against Nalm-6 cells.

ROS-mediated oxidative damage activating the autophagy signaling pathway can be protective or destructive (48). Autophagy is a recycling system considered a double-edged sword in cancer development with tumor suppressor and tumorigenic function. Therefore, ROSmediated activation of autophagy in cancer development is a great challenge in cancer research (48).

This study investigated several ATGs using qRT-PCR to verify whether RJ induces autophagy in cancer cells. BECN1, as a tumor suppressor gene, is a component of the phosphatidylinositol-3-kinase (PI3K) complex, which initiates the nucleation of phagophore and promotes the recruitment of other ATGs to phagosome formation (49). BECN1, ATG7, and ATG10 expression increased after Nalm-6 exposure with RJ. Therefore, RJ induced autophagy in BECN1 protein-mediated pathways. Additionally, RJ derivates could promote autophagy by activating the Unc-51-like autophagy-activating kinase (ULK) complex (50, 51). You *et al.* demonstrated that 10-HDA and 10-hydroxy decanoic acid (10-HDAA), as the fatty acid in RJ, promote autophagy via up-regulating the ULK-1 complex, which is evidenced by an elevated level of LC3-II, as a hallmark of autophagy, and decrease p62/SQSTM1 expression (50, 51). Hence, LC3-II and p62/SQSTM1 expression were examined to further explore the effect of autophagy activity of RJ. The results revealed a distinct increase in the LC3-II expression and decreased expression of p62/SQSTM1, consistent with autophagy activation. 

The activated autophagy is a compensatory mechanism for the anti-leukemic effect of RJ. Martínez-chacón and Paredes-barquero indicated that inhibition of 10-HAD-induced autophagy promotes neurotoxin 6-hydroxy-dopamine (6-OHDA) cytotoxicity against human dopaminergic neuroblastoma SH-SY5Y cells (52). Meanwhile, researchers showed that Torin-2 triggered autophagy potentially enhances the anti-cancer effect of Torin-2 on Nalm-6 cells (53). Conversely, Mehrpouri *et al.* revealed that cell viability diminished in Nalm-6 cells treated with CQ as an autophagy inhibitor (54). Consequently, the protective or destructive effect of RJ-induced autophagy in Nalm-6 cells remains elusive and is to be investigated in the future. Figure 7 provides an overview of plausible mechanisms by which RJ induces anti-leukemic effects in ALL-derived Nalm-6 cells.

## Conclusion

Based on the results, RJ is a natural product that can be taken orally, has no particular adverse reactions after consumption, and is a safe supplement for clinical use. This research shed new light on the anti-leukemic effect of RJ against ALL-derived Nalm-6 cells, which has never been reported in previous investigations. Overall, RJ inhibits metabolic activity, promotes intracellular ROS-mediated intrinsic apoptosis pathway, and decreases proliferative capacity, and triggers autophagy in Nalm-6 cells. Furthermore, the evaluation of the RJ effect on normal cells illustrated RJ being relatively non-toxic to normal cells, which improved the anti-leukemic activities of RJ against human ALL cells. 

Although this study reinforces the selective therapeutic potential of RJ, the significant limitations of this study were the lack of investigation of different characteristics of various RJ components and conducting the study only in the *in vitro* phase. Therefore, the effectiveness of RJ should be validated on ALL treatments. 

## Authors’ Contributions

S Y, M G, and A G designed the experiments; S Y, A NB, M I, and SZ H performed experiments and collected data; S Y and D B analyzed and interpreted the results, S Y, A NB, and D B wrote, reviewed, and edited the article; M B provided the graphical abstract and Figure 7; A G supervised, directed, and managed the study; S Y, D B, and A G approved the final version to be published. 

## Conflicts of Interest

The Authors have no conflicts of interest.
